# Complications After Deep Brain Stimulation: A 21-Year Experience in 426 Patients

**DOI:** 10.3389/fnagi.2022.819730

**Published:** 2022-04-07

**Authors:** In-Ho Jung, Kyung Won Chang, So Hee Park, Won Seok Chang, Hyun Ho Jung, Jin Woo Chang

**Affiliations:** ^1^Department of Neurosurgery, Brain Research Institute, Yonsei University College of Medicine, Seoul, South Korea; ^2^Department of Neurosurgery, Dankook University College of Medicine, Cheonan, South Korea

**Keywords:** deep brain stimulation, complication, Parkinson’s disease, essential tremor, dystonia, movement disorder, postoperative infection, intracerebral hemorrhage

## Abstract

**Background:**

Deep brain stimulation is an established treatment for movement disorders such as Parkinson’s disease, essential tremor, and dystonia. However, various complications that occur after deep brain stimulation are a major concern for patients and neurosurgeons.

**Objective:**

This study aimed to analyze various complications that occur after deep brain stimulation.

**Methods:**

We reviewed the medical records of patients with a movement disorder who underwent bilateral deep brain stimulation between 2000 and 2020. Among them, patients requiring revision surgery were analyzed.

**Results:**

A total of 426 patients underwent bilateral deep brain stimulation for a movement disorder. The primary disease was Parkinson’s disease in 315 patients, followed by dystonia in 71 patients and essential tremor in 40 patients. Twenty-six (6.1%) patients had complications requiring revision surgery; the most common complication was infection (12 patients, 2.8%).

**Conclusion:**

Various complications may occur after deep brain stimulation, and patient prognosis should be improved by reducing complications.

## Introduction

Deep brain stimulation (DBS) is non-destructive and reversible compared with ablative stereotactic neurosurgical procedures, such as thalamotomy and pallidotomy. Therefore, DBS is currently considered a conventional treatment for movement disorders, including Parkinson’s disease, essential tremor, and dystonia (Limousin et al., 1998; Schuurman et al., 2000; Simuni et al., 2002; Umemura et al., 2003; Okun, 2012; Larson, 2014; Krack et al., 2019).

However, various complications occurring after DBS are a major concern for patients and neurosurgeons (Umemura et al., 2003; Voges et al., 2006; Larson, 2014). For example, due to intracranial electrode insertion, DBS can cause intracerebral hemorrhage (ICH). In addition, DBS may cause skin erosion and infection because of the subcutaneous placement of a bulky foreign body called an internal pulse generator (IPG) and a long extension. The complications that require revision surgery after DBS exhaust patients physically, mentally, and economically (Chen et al., 2017).

Short-term studies with less than 4 years of mean follow-up on the complications occurring after DBS have been published, but no long-term follow-up studies have been conducted (Oh et al., 2002; Umemura et al., 2003; Lyons et al., 2004; Voges et al., 2006). Therefore, we aimed to conduct a large long-term study to review bilateral DBS performed by a single neurosurgeon at a single institution for 21 years. The complications that required revision surgery after DBS were analyzed, and our efforts to reduce the complication rate were described.

## Materials and Methods

Unilateral or bilateral DBS stimulation is performed for movement disorders (Parkinson’s disease, essential tremor, and dystonia), psychiatric disorders (Tourette’s syndrome and obsessive-compulsive disorder), and pain disorders. However, only patients with movement disorder who underwent bilateral DBS were included in this study to reduce bias due to various characteristics of the study subjects.

We selected the research subjects by reviewing medical records based on the following criteria: (1) patients who underwent bilateral DBS surgery (2) diagnosis of movement disorders such as Parkinson’s disease, essential tremor, and dystonia (3) DBS performed during the period from January 2000 to August 2020 and (4) postoperative follow-up period of more than 1 year.

Six hundred and ten patients who underwent DBS surgery between 2000 and 2010 were identified. A total of 109 patients underwent unilateral DBS, and 501 underwent bilateral DBS. Among the 501 patients, 450 had bilateral DBS for movement disorders, such as Parkinson’s disease, essential tremor, and dystonia. Twenty-four patients who were followed up for less than 1 year were excluded from the study. Four hundred and twenty-six patients who underwent bilateral DBS were finally enrolled and analyzed retrospectively.

Patients who underwent bilateral DBS surgery for movement disorders (Parkinson’s disease, essential tremor, and dystonia) between January 2000 and August 2020 and who had a follow-up period of more than 1 year were included in the study. All surgeries were performed by the same neurosurgeon (JWC).

We defined complications requiring revision surgery as an additional surgical treatment performed due to complications after DBS. In addition, complications after DBS surgery were divided into hardware- and surgery-related complications. Hardware-related complications were caused by device problems such as extension fracture or disconnection. Surgery-related complications were defined as complications caused by the surgical technique and included postoperative infection and ICH.

We divided the 426 cases of bilateral DBS surgery performed in the 21-year period into four chronological groups based on the operation date. Group A included 106 cases (March 2000–June 2007), group B, 106 cases (June 2007–March 2011), group C, 107 cases (April 2011–February 2017), and group D, 107 cases (March 2017–September 2020). The operation time, complication rate, and infection rate for each group were compared. Comparing these four chronological groups, it was possible to identify changes in surgical outcomes according to our institution’s surgical method and learning curve.

This study was conducted following the tenets of the Declaration of Helsinki and was approved by the Institutional Review Board of our institute (4-2021-1273). The requirement to obtain the patient’s written consent was waived as this was a retrospective study.

### Surgical Procedure

The process of mounting the stereotactic frame and the magnetic resonance imaging (MRI) for planning has been described in our previous report (Park et al., 2011; Kim et al., 2020; Jung et al., 2021). All patients were mounted on the Leksell stereotactic frame G (Elekta Instruments AB, Stockholm, Sweden) under local anesthesia on the day of the surgery. After the stereotactic frame was fixed to the head, the patients underwent an MRI evaluation (1.5 Tesla Philips Achieva). The MRI sequences for the stereotactic biopsy included gadolinium-enhanced T1-weighted images with a slice thickness of 1.5 mm and T2-weighted images with a slice thickness of 2.5 mm. The DBS surgery consists of two stages. The first stage is the insertion of an intracranial electrode, and the second stage is the insertion of an IPG. The first stage was usually performed with local anesthesia, but if the patient could not tolerate the intracranial procedure with local anesthesia, the procedure was performed under general anesthesia. After creating a burr hole, a 2–3-mm dura incision was made. The microTargeting Single Insertion Electrode (FHC, Inc., Bowdoin, ME, United States) was inserted as planned using the microTargeting Drive System (FHC, Inc.). An intracranial lead was inserted when the final electrode target was determined through a microelectrode recording. In patients with Parkinson’s disease, the target of the DBS electrode was the subthalamic nucleus or globus pallidus internus. In patients with essential tremor, the target was the ventralis intermedius nucleus, and in patients with dystonia, the target was the globus pallidus internus. When the first stage (i.e., insertion of the intracranial electrode) was completed, brain computed tomography (CT) was used to verify the accuracy of the DBS electrode placement and detect hemorrhage. The second stage was performed under general anesthesia, and an IPG was inserted into the anterior chest wall pocket above the pectoralis major muscle. After surgery, the condition of the device was checked using a cranial and chest X-ray.

### Statistical Analysis

Patients visited the outpatient clinic regularly to get an IPG check and medication adjustment. We identified the complications requiring revision operation during the follow-up period. The complications were divided into hardware- and surgery-related complications. The IBM SPSS Statistics software (version 25.0; IBM, Armonk, NY, United States) was used for the statistical analyses. The categorical variables were evaluated using the chi-square and Fisher’s exact tests. The continuous variables were evaluated using Student’s *T*-test, and the equality of variance was considered using Levene’s test. A *p*-value less than 0.05 was considered statistically significant.

## Results

Four hundred and twenty-six patients who underwent bilateral DBS were finally included and analyzed retrospectively. The analyzed patients comprised 227 (53.3%) women and 199 (46.7%) men. The mean age of the patients at the time of surgery was 57.5 years (range 8–77, standard deviation [SD] 11.9). The mean follow-up was 79.4 months (range 0–239, SD 54.6). The primary disease was Parkinson’s disease in 315 (73.9%) patients, followed by dystonia in 71 (16.7%) patients and essential tremor in 40 (9.4%) patients. The mean operation time was 287.2 min (range 135–581, SD 74.9), the mean blood loss was 19.8 mL (range 5–540, SD 49.3), and the mean patients’ body mass index (BMI) was 22.8 (range 12.6–41.4, SD 3.3).

The mortality rate was 0.5% (2/426). One patient died from aspiration pneumonia during the management of ICH and intraventricular hemorrhage (IVH) caused by intracranial electrode insertion. The other died from ventricular tachycardia during general anesthesia regardless of the surgical procedure. The morbidity rate was 1.4% (six patients; one intracranial abscess, two minor intracranial hemorrhages, and three major intracranial hemorrhages).

**FIGURE 1:**
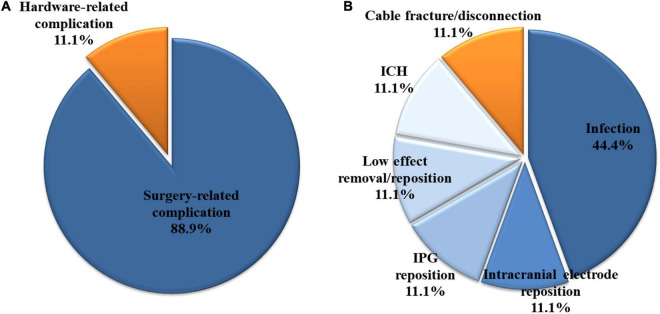
Among the 426 patients who underwent bilateral deep brain stimulation, 27 had complications requiring revision surgery. **(A)** Three (11.1%) and 24 (88.9%) of the 27 patients had hardware- and surgery-related complications, respectively. **(B)** All three hardware-related complications were impedance problems due to extension fracture or disconnection. Among the surgery-related complications, infection was the most common, accounting for 44.4% (12/27 patients) of all complications. ICH, intracerebral hemorrhage; IPG, internal pulse generator.

**FIGURE 2:**
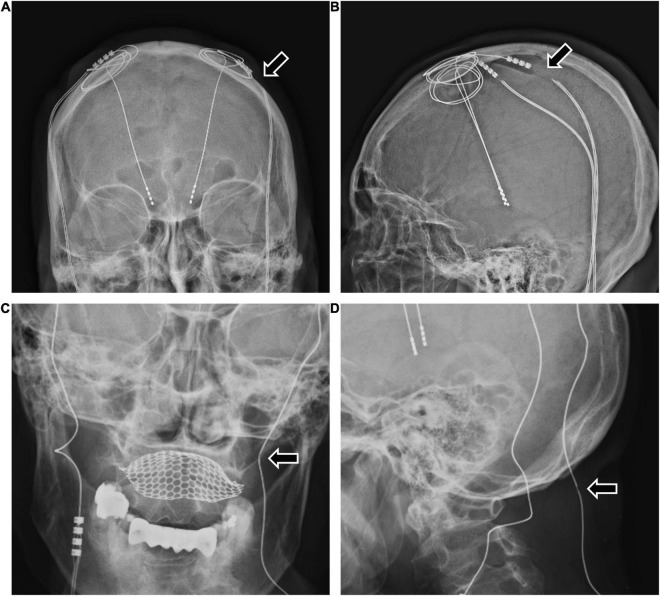
The two patients with bilateral DBS with high impedance due to extension wire fracture or disconnection. **(A,B)** A 63-year-old woman with Parkinson’s disease experienced sudden worsening of symptoms 3 years after bilateral DBS. The examination revealed high impedance of the patient’s left DBS system due to extension fracture (**A,B** arrows). After the revision operation to replace the extension wire, the patient’s DBS system was restored to normal function. **(C,D)** A 48-year-old woman with Parkinson’s disease experienced worsening symptoms 4 years after bilateral DBS. The examination revealed high impedance of the patient’s left DBS system and an extension wire abnormality (**C,D** arrows). After the revision operation to replace the electrode and the extension wire, the patient’s DBS system was restored to normal function. DBS, deep brain stimulation.

Among the 426 patients, 27 (6.3%) had complications requiring revision surgery. Three of the 27 patients had hardware-related complications, and 24 patients had surgery-related complications (Figure 1). All three hardware-related complications were impedance problems due to extension fracture or disconnection (Figures 1B, 2). The problems were solved by replacing the extensions. They occurred a minimum of 3 years after surgery (39.7 months, 48.7 months, and 116.3 months).

Among the surgery-related complications, infection was the most common, accounting for 44.4% (12/27 patients) of all complications. Most cases (11 patients) involved extracranial infections, but one case described a brain abscess located at the intracranial electrode. Of the 12 cases, infection occurred after the primary DBS operation and after battery change operation in eight and four cases, respectively. After the last operation, infection occurred after an average of 23.7 months (range 5.2–47.1, SD 14.7). Two of the 12 cases had infection after skin laceration due to trauma, and four cases had the wire exposed due to skin erosion. The characteristics of the 12 patients who developed infection are detailed in Table 1, and their characteristics were compared with those of the 414 patients who did not develop infection (Table 2). Sex, age, primary disease, operation time, blood loss, BMI, hypertension, and diabetes mellitus were not significantly different between the two groups. However, the BMI of patients who had infection tended to be lower than that of patients who had no infection (*p* = 0.080).

**TABLE 1:** Twelve cases of infection after deep brain stimulation.

Patient No.	1	2	3	4	5	6	7	8	9	10	11	12
Sex	M	M	M	M	F	M	M	F	M	F	F	F
Age at diagnosis	58	44	68	49	57	72	41	60	54	12	52	57
Last operation	DBS insertion	IPG change	DBS insertion	DBS insertion	DBS insertion	IPG change	DBS insertion	DBS insertion	DBS insertion	IPG change	IPG change	DBS insertion
Estimated blood loss (cc)	<10	<10	<10	<10	<10	<10	<10	<10	100	<10	<10	<10
Infection period (months)	13.2	40.9	36.3	47.1	34.1	26.0	9.5	13.3	37.0	12.6	9.8	5.2
Scalp laceration due to trauma					✓							✓
Skin erosion	✓	✓					✓	✓				
Microbiology culture	No growth	*Staphylococcus aureus*	*Pseudomonas aeruginosa*	No growth	*Staphylococcus aureus*	*Staphylococcus epidermidis*	*Staphylococcus aureus*	No growth	*Staphylococcus epidermidis*	*Staphylococcus epidermidis*	Staphylococcus, coagulase negative	*Staphylococcus aureus*
Device removal	Only debridement	Only debridement	Bilateral intracranial & IPG removal	Lt intracranial & IPG removal	Lt intracranial & IPG removal	Lt intracranial & IPG removal	Lt intracranial & IPG removal	Rt intracranial & IPG removal	Bilateral intracranial & IPG removal	Lt intracranial & IPG removal	Bilateral intracranial & IPG removal	Bilateral intracranial & IPG removal
BMI	18.4	24.7	21.1	19.3	18.2	23.9	25.3	21.9	24.7	12.6	18.9	24.7

*DBS, deep brain stimulation; IPG, internal pulse generator.*

**TABLE 2:** Characteristics of patients who developed infection after deep brain stimulation surgery.

	Normal cases	Cases with infection	*p*
Characteristics	(*N* = 414)	(*N* = 12)	
**Sex**			0.600
Female	222 (53.6%)	5 (41.7%)	
Male	192 (46.4%)	7 (58.3%)	
**Age** (y)	57.7 ± 11.7	52.0 ± 15.4	0.102
**Primary disease**			0.156
Parkinson’s disease	309 (74.6%)	6 (50.0%)	
Dystonia	67 (16.2%)	4 (33.3%)	
Essential tremor	38 (9.2%)	2 (16.7%)	
**Operation time** (min)	287.3 ± 74.6	285.9 ± 85.8	0.952
**Blood loss** (mL)	20.0 ± 49.8	12.9 ± 27.4	0.406
**BMI**	22.8 ± 3.3	21.1 ± 3.8	0.080
**Hypertension**	88 (21.3%)	4 (33.3%)	0.518
**Diabetes mellitus**	38 (9.2%)	1 (8.3%)	1.000

*BMI, body mass index.*

**FIGURE 3:**
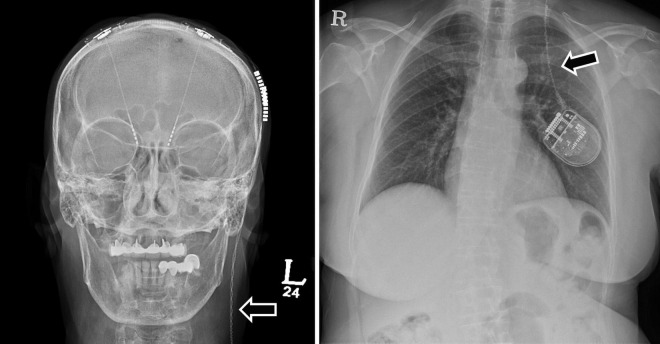
A 70-year-old woman with Parkinson’s disease had a broken extension wire due to Twiddler’s syndrome 3 months after primary DBS surgery. The skull and chest X-rays demonstrate twisted and broken extension wires due to Twiddler’s syndrome (arrows). The patient underwent revision surgery to replace the extension wire and perform multiple firm internal pulse generator anchor sutures. DBS, deep brain stimulation.

In surgery-related complications, DBS removal or intracranial electrode reinsertion was performed in six patients due to lower DBS effects than expected. IPG repositioning was also performed in three patients because of a problem in the interrogation and charging of the IPG. In one patient, the IPG was firmly fixed by revision surgery due to Twiddler’s syndrome (Figure 3). This syndrome is the conscious or subconscious manipulation of an IPG within its subcutaneous pocket, leading to twisting of extension wires and, ultimately, wire fracture.

**FIGURE 4:**
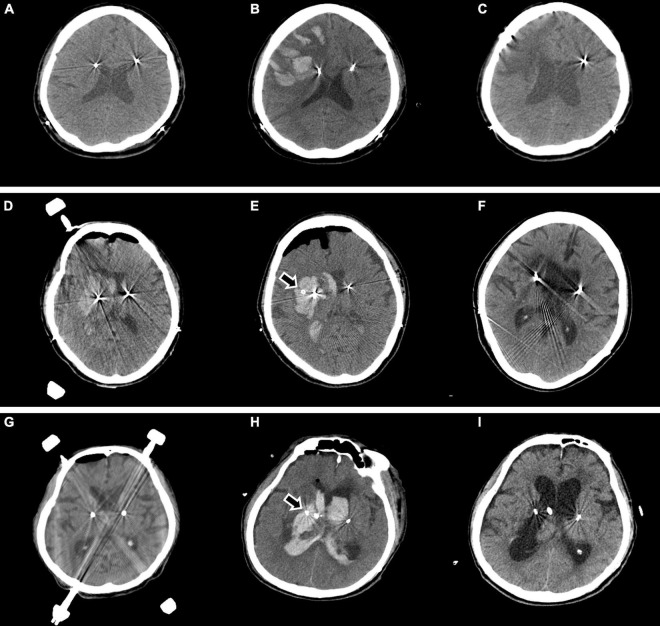
Major ICH requiring surgical treatment is observed in three cases. In one patient, no abnormalities are observed on brain CT immediately after surgery **(A)**, but ICH developed in the right frontal lobe with seizures 8 h after surgery **(B)**. The patient underwent craniotomy and hematoma evacuation due to a large ICH, increased edema, and a midline shift. Two months later, the patient was transferred for rehabilitation **(C)**. In another patient, right thalamic ICH and IVH are observed on brain CT after intracranial electrode insertion **(D)**, and hematoma catheter insertion was performed immediately (**E** arrow). After 1 month, the ICH was resolved, and the patient was transferred for rehabilitation **(F)**. In another patient, no abnormalities are observed on brain CT immediately after surgery **(G)**, but right thalamic ICH and IVH are observed on brain CT 16 h after the surgery, and hematoma catheter insertion was performed (**H** arrow). The hematoma is resolved **(I)**, but the patient died from aspiration pneumonia on postoperative day 30. ICH, intracerebral hemorrhage; CT, computed tomography; IVH, intraventricular hemorrhage.

Asymptomatic minor ICH that did not require surgical management occurred in four patients, and symptomatic minor ICH that did not require surgical management occurred in three patients. Major ICH requiring surgical treatment was observed in three patients. In one patient, no abnormalities were observed on brain CT immediately after surgery, but an ICH developed in the right frontal lobe with seizures 8 h after surgery. The patient underwent craniotomy and hematoma evacuation due to the large ICH, increased edema, and a midline shift. The patient was transferred for rehabilitation (Figures 4A–C). In another patient, right thalamic ICH and IVH were observed on the brain CT after intracranial electrode insertion, and a hematoma catheter insertion was performed immediately. After 1 month, the ICH was resolved, and the patient was transferred for rehabilitation (Figures 4D–F). Finally, in one patient, no abnormalities were observed on the brain CT immediately after surgery, but right thalamic ICH and IVH were observed on brain CT 16 h after the operation, and a hematoma catheter insertion was performed. The hematoma was resolved, but the patient died from aspiration pneumonia on postoperative day 30 (Figures 4G–I).

The characteristics of the 10 patients who developed ICH were compared with those of 416 patients who did not develop ICH (Table 3). Sex, age, primary disease, operation time, blood loss, BMI, hypertension, and diabetes mellitus were not significantly different between the two groups.

**TABLE 3:** Characteristics of patients who developed intracerebral hemorrhage after deep brain stimulation surgery.

	Normal case	Case with ICH	*p*
Characteristics	(*N* = 416)	(*N* = 10)	
**Sex**			0.756
Female	221 (53.1%)	6 (60.0%)	
Male	195 (46.9%)	4 (40.0%)	
**Age** (y)	57.5 ± 11.9	60.1 ± 9.1	0.488
**Primary disease**			0.644
Parkinson’s disease	306 (73.6%)	9 (90.0%)	
Dystonia	70 (16.8%)	1 (10.0%)	
Essential tremor	40 (9.6%)	0 (0.0%)	
**Operation time** (min)	288.2 ± 74.7	245.5 ± 74.5	0.075
**Blood loss** (mL)	19.3 ± 47.8	41.0 ± 95.0	0.490
**BMI**	22.8 ± 3.3	23.0 ± 3.2	0.827
**Hypertension**	88 (21.2%)	4 (40.0%)	0.233
**Diabetes mellitus**	37 (8.9%)	2 (20.0%)	0.231

*ICH, intracerebral hemorrhage; BMI: body mass index.*

**FIGURE 5:**
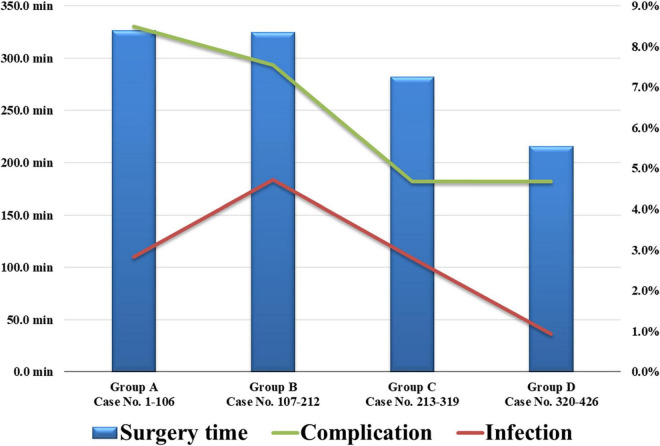
The 426 cases of bilateral DBS surgery were divided into four chronological groups based on their operation date. The recent average operation time and complications and infections rates were reduced. DBS, deep brain stimulation.

We divided the 426 cases of bilateral DBS surgery performed for 21 years into four groups based on the operation date. Group A included 106 cases operated between March 2000 and June 2007; group B, 106 cases operated between June 2007 and March 2011; group C, 107 cases operated between April 2011 and February 2017; and group D, 107 cases operated between March 2017 and September 2020. The operation time, complication rate, and infection rate for each group are described in Figure 5. The average operation time initially exceeded 300 min, but recently, with the accumulated experience, the average operation time had been reduced to < 200 min. In addition, the recent complications and infections rates were also lower than those in the early days of DBS surgery.

## Discussion

Complications that can occur after DBS surgery are diverse, and these severely affect doctors and patients. Among the 426 patients with movement disorders who underwent bilateral DBS insertion, 27 (6.3%) patients had complications requiring revision operation. Infection was the cause of the complication in 12 patients. The infection rate was 2.8%, lower than the 2.95–6.2% reported in previous studies (Umemura et al., 2003; Lyons et al., 2004; Voges et al., 2006; Pepper et al., 2013; Tolleson et al., 2014; Chen et al., 2017; Doshi et al., 2021). Infection after DBS surgery is the most frustrating complication because, in many cases, all devices inserted during the primary operation must be removed (Voges et al., 2006; Chen et al., 2017; Kim et al., 2020). The DBS system is physically connected from the intracranial electrode to the IPG by an extension wire. Once an infection occurs, the bacteria spread readily along the wire. Therefore, infection after DBS surgery cannot be resolved with only local debridement without device removal. Among the 12 cases with infection, only two were resolved with simple debridement and antibiotics without device removal. In six of the remaining 10 cases, the extension wire, IPG, and an intracranial electrode on the side of the infection were all removed. In the remaining four cases, the infection was resolved after removing the infected side and the opposite site device.

Short-term follow-up studies with less than 4 years of mean follow-up of complications occurring after DBS have been published (Oh et al., 2002; Umemura et al., 2003; Lyons et al., 2004; Voges et al., 2006), but no long-term follow-up studies have been conducted. Our study had a mean follow-up period of 79.4 months (6.6 years). Therefore, we consider that our study has an adequate power due to its larger size and follow-up duration than previous studies. In addition, the postoperative infection of the 12 patients developed at an average of 23.8 months (5.2–47.1 months) after the DBS surgery. It is noteworthy that no infection was identified after 4 years post-surgery. Hence, our investigation has a longer follow-up period than the previous ones, but the complication rate is not higher.

In six of the 12 cases, infection occurred after external environment connection, e.g., scalp laceration and skin erosion. Defects of the skin barrier are a common finding in patients with infection after DBS surgery because they provide an entrance for causative bacteria (Oh et al., 2002; Kenney et al., 2007; Sillay et al., 2008; Fily et al., 2011). Therefore, patients with DBS should avoid trauma-related laceration, and neurosurgeons should perform subcutaneous tunneling of extension wires at an appropriate depth during surgery. Skin erosion may occur later if the tunneling at a subcutaneous level is too shallow and close to the skin. We predicted that people with low BMI have a thin subcutaneous fat layer, so the extension passes close to the skin, increasing the occurrence of skin erosion and infection. However, our prediction did not achieve statistical significance in this study but only a statistical tendency. In patients with a thin subcutaneous fat layer, additional small skin incisions in the retro auricular or peri clavicle areas could help in subcutaneous tunneling at the appropriate depth.

Although statistical significance was not reached in the present study, many studies had reported that operation time and blood loss affect postoperative infection in DBS surgery (Ogihara et al., 2009; Wong et al., 2012; Tolleson et al., 2014). Therefore, we attempted to minimize operation time and blood loss in DBS surgery. The average operation time at our institution was 326.7 min in the beginning, but recently, it has been greatly reduced to 215.5 min. The primary reason for the shortened surgery time is the application of a single subcutaneous tunneling and a single battery insertion by using one double-channel battery instead of two single-channel batteries in bilateral DBS surgery since 2017. The second reason is using intraoperative CT, performed in our institution since 2018. Previously, after the first stage of intracranial electrode insertion, the patient had to leave the operating room and move to the CT room to verify electrode location. After the introduction of intraoperative CT, performing CT scans in the operating room allowed shortening of the operation time. The last reason is the accumulation of experience. This includes advances in the skills of the neurosurgeon, such as skin incision, drilling, and suture, and the rapid assessment of microelectrode recording when inserting intracranial electrodes. In addition, fast and accurate reading of the microelectrode recording is important for the proper placement of the intracranial electrode (Kocabicak et al., 2019; Soares et al., 2019; Reddy et al., 2020). We consider that the accumulation of experience and the technological advances in DBS surgery could shorten the operation time and reduce the complications and infections rates.

Intracerebral hemorrhage caused by intracranial electrode insertion is the most fatal among the complications after the DBS surgery; it is the most common cause of mortality and morbidity in DBS surgery. In this study, asymptomatic minor ICH that did not require surgical management occurred in four patients, symptomatic minor ICH that did not require surgical management occurred in three patients, and major ICH that required surgical management occurred in three patients. After the DBS surgery, the incidence of all ICH is 2.3%, and the incidence of a major ICH is 0.7%. In previous investigations, the incidence of an ICH after DBS surgery was 2.5–5.1% (Schuurman et al., 2000; Oh et al., 2002; Fenoy and Simpson<suffix>Jr.</suffix>, 2014; Falowski et al., 2015; Park et al., 2017). Since the target location is fixed in DBS surgery, neurosurgery can modify the intracranial electrode trajectory by moving the entry point. Therefore, the neurosurgeon should carefully analyze the preoperative MRI to establish a trajectory to avoid arteries and veins. Although hypertension was not significantly higher in patients who developed ICH after DBS surgery in this study, previous investigations reported hypertension as a risk factor for ICH after DBS surgery. Voges et al. (2007) found that arterial hypertension and coagulopathy are risk factors for ICH after DBS surgery in patients aged ≥ 60 years. Yang et al. (2020) reported that male sex and hypertension are risk factors for ICH after DBS in Parkinson’s disease. Two of the three patients with major ICH had no specific findings on immediate postoperative CT, but an ICH was observed on follow-up CT within 16 h of surgery. Therefore, we recommend careful blood pressure control during DBS surgery and up to 1 day after surgery.

Apart from infection and ICH, surgery-related complications include IPG repositioning, removal due to low therapeutic effect, and intracranial electrode repositioning.

Internal pulse generator repositioning was performed in three cases due to interrogation or recharging problems. This problem was not due to a defective device but due to an improper fixation of the IPG. Therefore, we classified these three cases of IPG repositioning as surgery-related complications and not hardware-related complications. IPG repositioning causes discomfort to the patient and may cause the IPG to overturn, thus interfering with the interrogation and recharging of the IPG. In addition, the continuation of IPG movement is the cause of the Twiddler’s syndrome mentioned earlier. The Twiddler’s syndrome was first described in patients with pacemakers and has been occasionally reported in patients with DBS (Burdick et al., 2010; Gelabert-Gonzalez et al., 2010; Penn et al., 2012; Franzini et al., 2018; Jackowiak et al., 2019; Adams and Shivkumar, 2020). This syndrome occurs because of the manipulation of the IPG within its subcutaneous pocket by the patient, leading to twisting of the extension wires and eventually an extension wire fracture. In previous studies, loose subcutaneous tissue, obesity, old age, obsessive-compulsive behavior, large subcutaneous pockets, and inappropriate IPG fixation were reported as predisposing factors for Twiddler’s syndrome (Machado et al., 2005; Geissinger and Neal, 2007; Israel and Spivak, 2008; Burdick et al., 2010; Gelabert-Gonzalez et al., 2010; Astradsson et al., 2011; Silva et al., 2014; Sobstyl et al., 2015, 2017). Neurosurgeons cannot change patient characteristics such as subcutaneous tissue, age, and behavior. However, the IPG repositioning revision surgery incidence can be reduced by creating an appropriately sized subcutaneous pocket and performing firm multiple suture anchors.

Low therapeutic effect removal was performed in three cases. The device was removed or repositioned when the patient’s symptoms did not improve despite the precise placement of the intracranial electrode in the planned location. The incidence of these complications requiring revision surgery may be reduced through a more careful patient selection and better preoperative target planning.

There were three cases where intracranial electrode repositioning was performed due to displacement of the intracranial electrode from our planned location. These complications can be reduced if the neurosurgeon fixes the electrode firmly and prevents the electrode from moving during skin and subcutaneous sutures.

Hardware-related complications occurred in three patients, including fracture or disconnection of extension wires. Normal DBS function was restored in all three patients by replacing the defective extension wire. Hardware-related complications accounted for a small proportion of the complications requiring revision surgery after DBS (3/27, 11.1%). We expect that advances in technology and improvement of DBS devices will further lower the hardware-related complication rate. All hardware-related complications occurred before 2010, and none occurred after 2010.

Our study is limited by its retrospective study design. In addition, since the number of infection cases (12 cases) and ICH cases (10 cases) was small, there may be a bias in statistical analyses identifying their characteristics. Therefore, caution should be exercised in the interpretation of the statistical analyses.

In conclusion, various complications can occur after DBS surgery. Twenty-six (6.1%) patients had complications requiring revision surgery, with the most common complication being infection (12 patients, 2.8%). These complications worsen the patient’s prognosis. We attempted through this study to share our experience in the management of DBS complications and our efforts to reduce them. We believe that our experience is potentially helpful for neurosurgeons performing DBS surgery.

## Data Availability Statement

The raw data supporting the conclusions of this article will be made available by the authors, without undue reservation.

## Ethics Statement

The studies involving human participants were reviewed and approved by the Institutional Review Board of Severance Hospital. Written informed consent for participation was not required for this study in accordance with the national legislation and the institutional requirements.

## Author Contributions

I-HJ: original draft, data curation, formal analysis, and methodology. KC and SP: data curation and formal analysis. WC and HJ: review and editing. JC: supervision, resources, review, and editing. All authors contributed to the article and approved the submitted version.

## Conflict of Interest

The authors declare that the research was conducted in the absence of any commercial or financial relationships that could be construed as a potential conflict of interest.

## Publisher’s Note

All claims expressed in this article are solely those of the authors and do not necessarily represent those of their affiliated organizations, or those of the publisher, the editors and the reviewers. Any product that may be evaluated in this article, or claim that may be made by its manufacturer, is not guaranteed or endorsed by the publisher.
